# ZONAB Regulates DNA Methylation, Mitochondrial Function, and Entry into Cell Senescence of Endothelial Cells

**DOI:** 10.3390/cells15111015

**Published:** 2026-05-31

**Authors:** Wenyi Jiang, Eleanor Lynam, Juliette Delafosse, Graeme M. Birdsey, Anna M. Randi, Karl Matter, Maria S. Balda

**Affiliations:** 1 UCL Institute of Ophthalmology, University College London, London EC1V 9EL, UK; 2 National Heart and Lung Institute (NHLI), Imperial College London, London W12 0NN, UK

**Keywords:** YBX3, angiogenesis, epigenetic, cell cycle, mitochondria, oxidative stress, oxidative phosphorylation, senescence

## Abstract

**Highlights:**

**What are the main findings?**

- The Y-box factor ZONAB/Ybx3 regulates DNA methylation and genome-wide expression of genes controlling the cell cycle, entry into cellular senescence in endothelia, and angiogenesis.
- Inactivation of ZONAB leads to increased mitochondrial fragmentation, decreased mitochondrial capacity, and increased ROS.

**What are the implications of the main findings?**

- ZONAB regulates endothelial homeostasis.
- ZONAB’s role in regulating endothelial energy metabolism and ROS signalling is compatible with a role in vascular health and correlates with the association of its gene with risk loci for vascular disorders.

**Abstract:**

Regulation of the endothelial stress response is important for blood vessel homeostasis and angiogenesis, processes disrupted in common vascular diseases and ageing. Here, we discovered that the Y-box factor ZONAB (ZO-1-associated nucleic acid binding protein; *YBX3*), a gene associated with risk loci for severe vascular disorders, regulates endothelial homeostasis and angiogenesis. By combining cell-based assays with primary endothelial cells and genome-wide expression and methylation measurements, we found that ZONAB depletion results in mitochondrial deregulation, increased reactive oxygen species, and a defective oxidative stress response, which correlates with increased promoter methylation of cell cycle genes. ZONAB depletion triggered cellular senescence via a phosphatidylinositol 3-kinase (PI3K)/Akt-dependent pathway, which was attenuated by PIK3 inhibitors, an antioxidant, or by drugs targeting mitochondrial function or fragmentation. Thus, our results reveal that ZONAB repression in endothelial cells leads to genome-wide changes in gene expression and DNA methylation, regulating endothelial proliferation and inflammation, as well as mitochondrial deregulation to promote cellular senescence. Hence, ZONAB supports endothelial homeostasis and may play a role in vascular health.

## 1. Introduction

Endothelial cells line the internal walls of blood and lymphatic vessels. They can exhibit high plasticity and can switch between a quiescent state, which supports vessel stability and function, and a proliferative and migratory state required for angiogenesis. Both angiogenesis and vessel homeostasis are affected by ageing, which generally results in a decrease in angiogenic potential and an increase in rates of diseases induced by endothelial malfunction, such as atherosclerosis and microvascular conditions affecting the heart and/or the brain [1,2]. Endothelial senescence induced by ageing and chronic stress is a key driver of such diseases, indicating that mechanisms that support the endothelial stress response are crucial for preventing the induction of stress-induced senescence. However, these mechanisms are only partially understood.

Cellular senescence is a state of permanent stop in cell proliferation induced by ageing or stress. It may be stimulated by telomere shortening, loss of telomere functionality, or by stress conditions, such as oxidative or oncogenic stress [2,3]. Accumulation of senescent cells often induces an inflammatory environment due to the release of proinflammatory stimuli such as IL1α/β and IL6 by the senescent cells, a process called the senescence-associated secretory phenotype (SASP). While SASP promotes processes such as wound healing, the accumulation of senescent cells with a SASP in ageing stimulates chronic inflammatory conditions due to the continuous release of proinflammatory factors.

Given the importance of stress for the induction of senescence, one would expect that genetic variants of stress response genes exist that predispose people to develop diseases such as atherosclerosis. Genome-wide association studies (GWASs) focusing on atherosclerosis identified such genes based on their association with loci that increase the risk of developing the disease [4,5]. Similarly, another study focused on genetic variants predisposing people to high systolic blood pressure, also a process affected by endothelial homeostasis [6]. A common gene identified by those studies is *YBX3*. Variants in this gene have also been linked to ovarian ageing [7], suggesting that its function could be linked to tissue ageing and cellular senescence.

*YBX3* encodes a multifunctional nucleic acid-binding protein that is also called ZONAB (ZO-1-associated nucleic acid binding protein), as it can interact with the tight junction protein ZO-1 [8,9]. In epithelial cells, ZONAB controls cell density and proliferation, and the cellular stress response [9,10,11,12,13]. ZONAB controls G1/S phase transition by transcriptional regulation of cyclin D1 and PCNA, and by forming a complex with CDK4 [9,11]. ZONAB is not only regulated by binding to ZO-1 but is also inhibited by Ral A, a Ras-effector pathway, and activated by the RhoA activator GEF-H1 [10,13,14]. Overexpression of ZONAB has been linked to cancer cell proliferation and metastasis in multiple tissues. However, the underlying mechanisms for its role in tumorigenesis are not well defined [15,16,17,18,19]. These studies have generally been performed in immortalised epithelial cell lines; therefore, whether loss of ZONAB function is linked to cellular senescence and if it is functionally relevant in endothelia remains unknown.

Here, we show that ZONAB is required for angiogenesis in vivo and normal angiogenic properties in vitro. ZONAB depletion in human primary endothelial cells induced cellular senescence. Cellular assays and genome-wide expression and methylation analyses indicate that ZONAB depletion resulted in changes in gene expression and methylation-driven epigenetic changes, and led to mitochondrial fragmentation and dysfunction, a disrupted oxidative stress response, and cellular senescence. Both inhibition of mitochondrial fragmentation and treatment with an antioxidant inhibited induction of cell senescence, suggesting that dysregulation of mitochondria and oxidative stress are key consequences of ZONAB malfunction.

## 2. Materials and Methods

### 2.1. Cell Culture, Transfection, and Transendothelial Electrical Resistance

**Figure 1:**
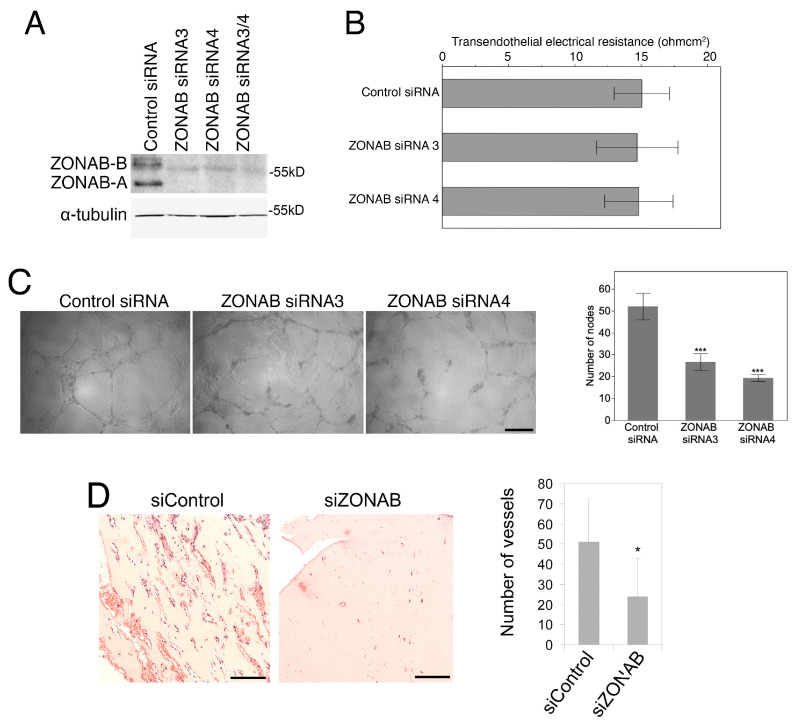
ZONAB regulates endothelial cell migration and angiogenesis. (**A**) Analysis of ZONAB expression by immunoblotting of cell lysates of HDMECs transfected with non-targeting control siRNAs or with two different ZONAB-targeting siRNAs (ZONAB siRNA 3 or 4) or as a pool (ZONAB siRNA 3/4). (**B**) Transendothelial electrical resistance was measured by ECIS at 500Hz. Means ± 1 SD, *n*= 4. (**C**) HDMECs were transfected with the indicated siRNAs and, 48 h later, replated on 100% Matrigel to test their angiogenic potential in a tube formation assay on Matrigel. Network formation was analysed after 16 h. The number of branches in each sample was quantified. Means ± 1 SD, *n* = 3. (**D**) The effect of ZONAB depletion on angiogenesis in vivo was measured by injecting mice with Matrigel-containing FGF and siRNAs as indicated to induce angiogenesis. After 7 days, the plugs were harvested and analysed. Means ± 1 SD; control siRNA, *n* = 6; ZONAB siRNA, *n* = 12). Bars, 250 μm. *t*-tests: * *p* < 0.05, *** *p* < 0.001.

**Figure 2:**
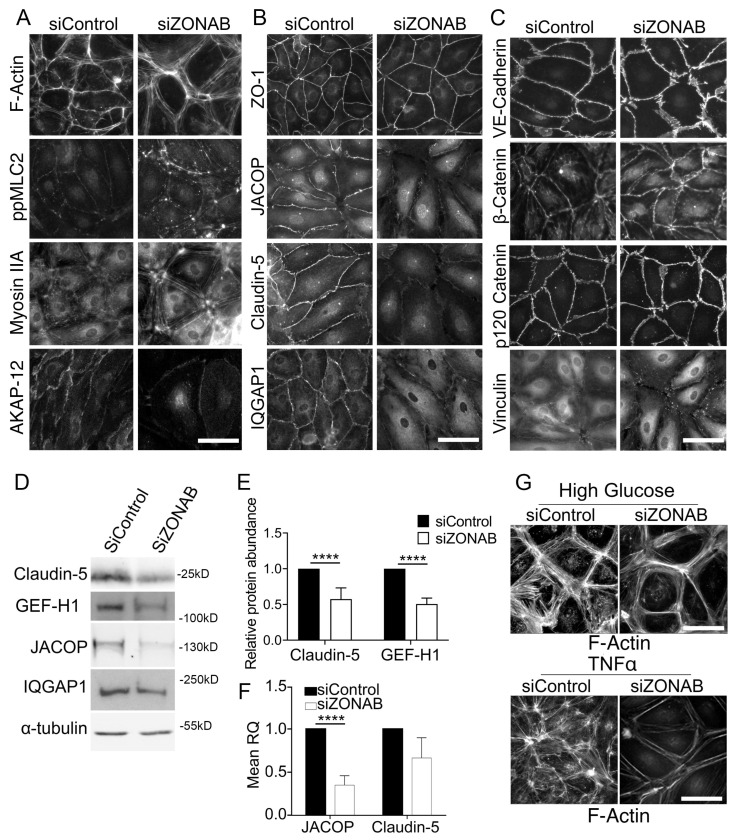
ZONAB depletion affects the actomyosin cytoskeleton and junctional complexes. (**A**–**C**) Immunofluorescence images of HDMECs treated with control or ZONAB siRNAs for 72 h before immunofluorescent staining with phalloidin for the visualisation of F-actin or antibodies against the indicated proteins. The JACOP images in panel (**B**) and the vinculin images in panel C include images from different channels acquired from the same microscopic fields in double-immunofluorescence experiments; therefore, cell outlines are expected to overlap. Scale bar, 50 μm. (**D**,**E**) Immunoblotting of lysates from cells transfected with siRNAs for 72 h. α-tubulin (bottom) was used as a loading control for the densitometric analysis shown in panel (**E**). (**F**) mRNA expression analysis by RT-qPCR for JACOP and claudin-5 in control or ZONAB siRNA-treated cells. Values were normalised using GAPDH expression, and obtained ratios were normalised to control siRNA values (means ± 1 SD, *n* = 3). (**G**) F-actin staining of HDMECs transfected with control or ZONAB siRNA in medium with high glucose (33 mM, for 24 h) or stimulated with 5 ng/mL TNFα for 5 h. Scale bar: 50 μm. All graphs show means ± 1 SD (*n* = 3). *t*-tests: **** *p* ≤ 0.0001.

**Figure 3:**
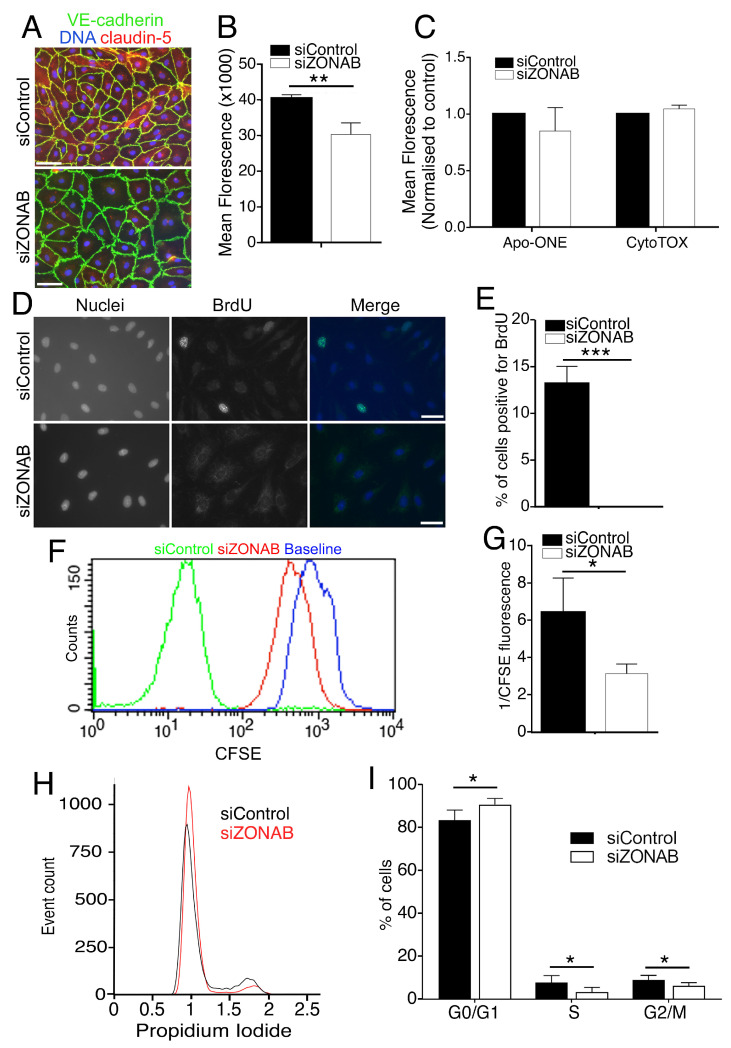
ZONAB regulates endothelial cell proliferation. (**A**) Immunofluorescence of siRNA-transfected HDMECs stained for VE-cadherin, claudin-5, and nuclei. Scale bar, 50 μm. (**B**) Cell density was assessed by measuring DNA content using the CyQUANT assay. (**C**) Caspase-3/7 activity (Apo-ONE) and LDH release (CytoTox) measurements of siRNA-transfected cells. Values were normalised to control siRNA determinations. (**D**,**E**) Assessment of proliferation by BrdU incorporation. Shown are images of nuclear and BrdU staining. Scale bar: 50 μm. (**F**,**G**) Cell division capacity assessed by CFSE CellTrace tracking dye. (**H**) Propidium iodide flow cytometric analysis of cell cycle stages. (**I**) The quantification shows the percentage of cells in G0/G1, S, and M phases. All graphs show means ± 1 SD (*n* = 3). *t*-tests: * *p* ≤ 0.05, ** *p* ≤ 0.01, *** *p* ≤ 0.001.

**Figure 4:**
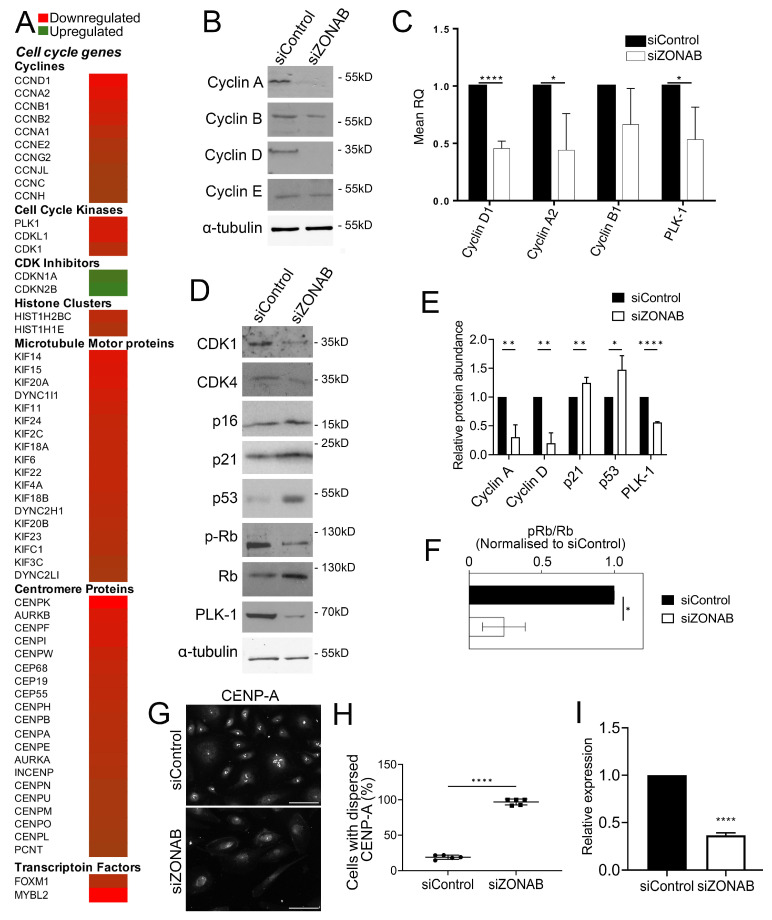
ZONAB depletion reduces the expression of cell cycle genes. (**A**) Expression analysis by RNA sequencing of control and ZONAB siRNA-transfected cells. Three mRNA isolations were analysed for each siRNA set. Shown are genes repressed or induced by ZONAB depletion (change in expression log_2_ > |0.5|; see also Tables S2 and S7). (**B**,**D**,**E**) Expression of the indicated cell cycle regulators was analysed by immunoblotting. α-tubulin was used as a loading control for the quantifications shown in panel (**E**) (means ± 1 SD, *n* = 3). (**C**) RT-qPCR for mRNA quantification of selected cell cycle regulators. Values were normalised using GAPDH expression, and obtained ratios were normalised to control siRNA values (means ± 1 SD, *n* = 3). (**F**,**G**) Immunofluorescent staining for CENP-A. Scale bar: 100 μm, the quantification shows percentage of cells with dispersed staining for CENP-A (means ± 1 SD, *n* = 5 images from 3 independent experiments). (**H**) Quantification of cells with dispersed CENPA-A. (**I**) mRNA expression of CENP-A in control or ZONAB siRNA-transfected cells (means ± 1 SD, *n* = 3). *t*-tests: * *p* < 0.05, ** *p* < 0.01, **** *p* < 0.0001.

**Figure 5:**
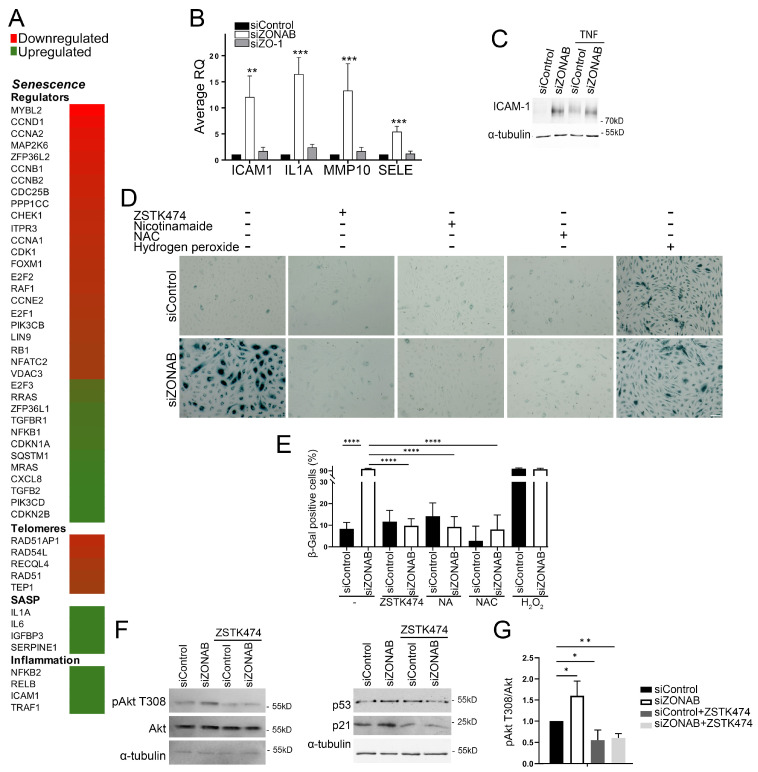
ZONAB depletion induces a senescent inflammatory phenotype that can be rescued by inhibition of PI3K. (**A**) Repression or induction of genes associated with cellular senescence, telomeres, the senescence-associated secretory phenotype (SASP), and endothelial inflammation (change in expression log_2_ > |0.5|; see also Tables S2 and S7). (**B**) RT-qPCR analysis of genes associated with inflammation. Values were normalised using GAPDH expression, and obtained ratios were normalised to control siRNA values (means ± 1 SD, *n* = 3). (**C**) Immunoblot of ICAM-1 expression upon ZONAB depletion without or with TNFα treatment. (**D**,**E**) Senescence-associated β-Galactosidase (SAβG) staining of siRNA-transfected cells without or with incubation for 48 h with 1 μM PI3K inhibitor ZSTK474, 200 μM nicotinamide, or 8 mM NAC. A positive control was incubated for 6 h with 150 μM H_2_O_2_. Scale bar: 200 μm. Cells were imaged by brightfield microscopy, and SAβG-positive cells were counted (panel (**E**), means ± 1 SD, *n* = 3). (**F**,**G**) Immunoblotting for pAkt T308 and Akt, as well as for p53 and p21 shows that PI3K inhibition effectively represses Akt phosphorylation and expression of p21 and p53. The quantification in panel (**G**) shows means ± 1 SD (*n* = 3). Note, phosphorylation of S473 was not affected by ZONAB depletion (Figure S2). *t*-tests: * *p* < 0.05, ** *p* < 0.01, *** *p* < 0.001, **** *p* < 0.0001.

**Figure 6:**
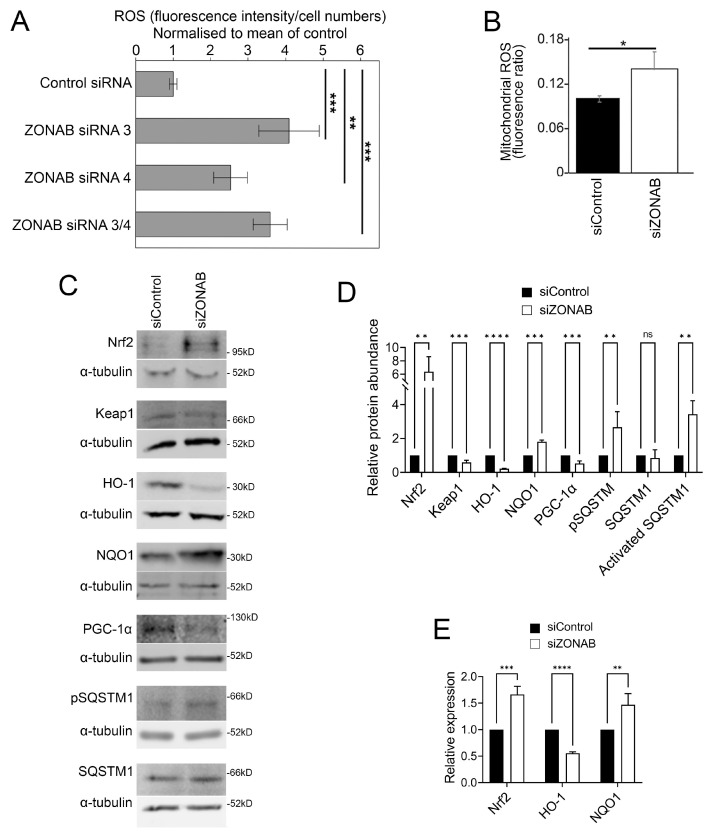
ZONAB deficiency leads to increased ROS levels. (**A**) Determination of cellular ROS levels with DCFHDA (means ± 1 SD, *n* = 4). (**B**) ZONAB depletion induces increased mitochondrial ROS as determined by incubating cells with reduced and oxidised forms of MitoTracker Orange CMTMRos (means ± 1 SD, *n* = 3). (**C**) Immunoblotting and (**D**) densitometric quantification for indicated oxidative stress response genes. α-Tubulin was used as a loading control (means ± 1 SD, *n* = 3). (**E**) RT-qPCR analysis of oxidative stress response genes. Values were normalised using GAPDH expression, and obtained ratios were normalised to control siRNA values (means ± 1 SD, *n* = 3). (**A**), Tukey–Kramer HSD test; (**B**–**E**), *t*-tests; * *p* < 0.05, ** *p* < 0.01, *** *p* < 0.001, **** *p* < 0.0001. *n* is the number of biological repeats used for the analysis.

**Figure 7:**
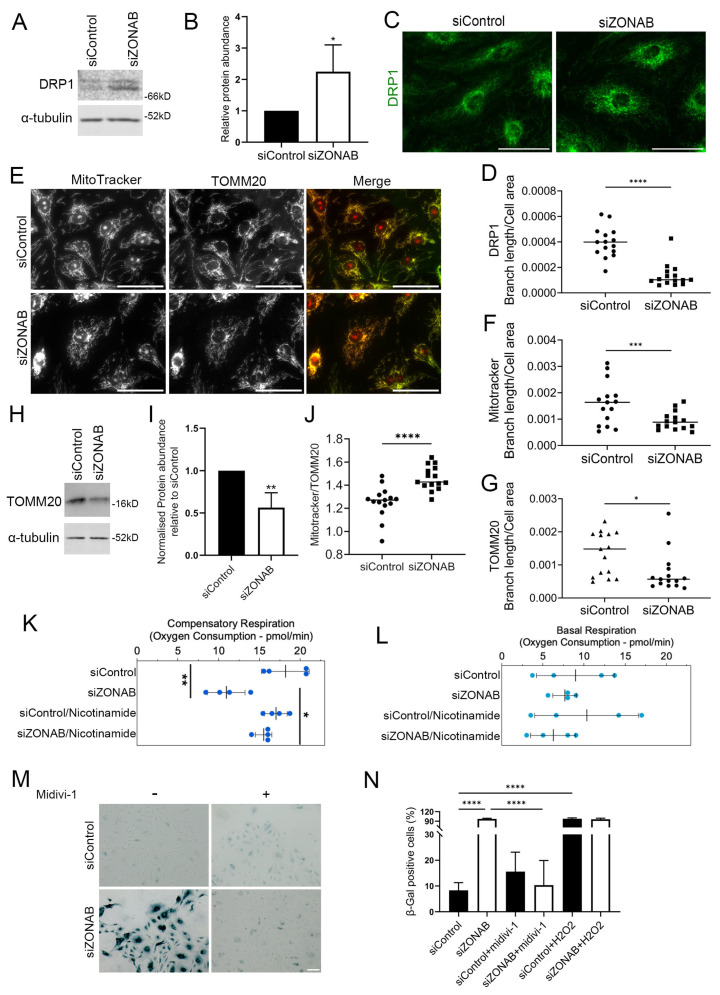
ZONAB deficiency leads to mitochondrial dysfunction. (**A**,**B**) Effect of ZONAB depletion on DRP1 expression assessed by immunoblotting. α-tubulin was used as a loading control in the densitometric quantification in panel B (means ± 1 SD, *n* = 3). (**C**,**D**) Immunofluorescence staining of DRP1 indicates increased mitochondrial fragmentation (scale bar: 50 μm; see Figure S3 for higher-resolution images). The quantification in panel (**D**) shows branch length relative to the cell area of the DRP1 staining (shown are means and datapoints, *n* = 15). (**E**) Images of TOMM20 immunofluorescence and MitoTracker staining in control or ZONAB siRNA-transfected HDMECs (scale bar: 50 μm; see Figure S3 for higher resolution images). Panels (**F**,**G**) show a quantification of the branch length for MitoTracker (**F**) and TOMM20 (**G**) relative to the cell area. Panel (**J**) shows the ratio of MitoTracker to TOMM20 staining. All three quantifications show means and data points (*n* = 15 images from 3 independent experiments). (**H**,**I**) Immunoblotting of TOMM20. Panel (**I**) shows a densitometric quantification of the immunoblots using α-tubulin as a loading control for normalisation. (**K**,**L**) Measurements of compensatory and basal respiration using a mitochondrial stress test protocol combined with a Seahorse analyser (shown are means ± 1 SD and datapoints, *n* = 4). (**M**,**N**) SAβG staining of control and ZOANB siRNA-transfected cells after a 48 h incubation without or with DRP1 inhibitor midivi-1. Scale bar: 200 μm. β-gal positive cells were imaged by bright field microscopy and quantified (panel (**H**), means ± 1 SD, *n* = 3). Panels (**B**,**D**,**F**,**G**,**J**): *t*-tests. Panels (**K**,**L**,**N**): Tukey HSD. * *p* < 0.05, ** *p* < 0.01, *** *p* < 0.001, **** *p* < 0.0001.

**Figure 8:**
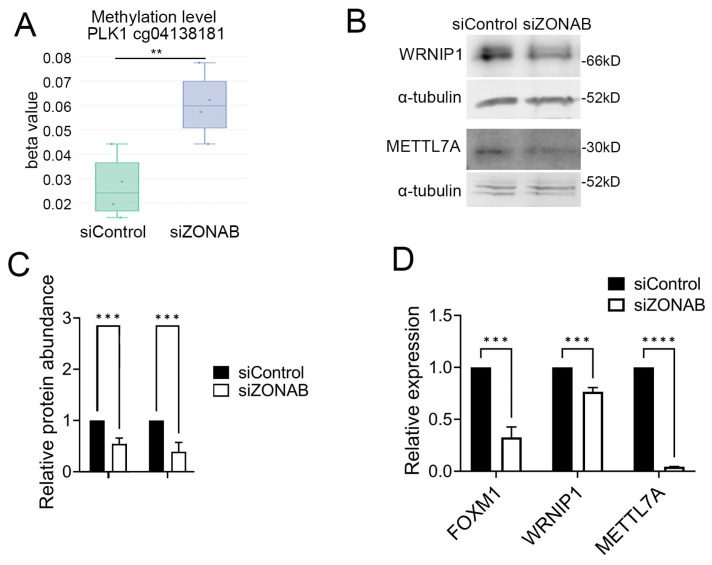
ZONAB regulates DNA methylation of genes involved in endothelial cell proliferation and senescence. (**A**) Boxplot analysis of the DNA methylation status of the PLK1 promoter at cg04138181 from the Illumina EPIC methylation array (*n* = 4; median, centre line; inter-quartile range, box; 95th percentiles, whisker). See Table S9 for examples of other genes identified as being differentially methylated. (**B**,**C**) Immunoblotting reveals repression of WRNIP1 and METTL7A. Densitometric quantification of at least 3 repeat experiments. Values were normalised to α-tubulin expression, and ratios for control siRNA-transfected samples were set to 1 (means ± 1 SD). (**D**) Analysis of mRNA expression of FOXM1, WRNIP1, and METTL7A relative to GAPDH determined by RT-qPCR (means ± 1 SD, *n* = 3). *t*-tests: ** *p* < 0.01, *** *p* < 0.001, **** *p* < 0.0001.

Human dermal microvascular endothelial cells (HDMEC adult C-12212 from 18–24-year-old donors and juvenile C-12210) were obtained from Promo Cell (Heidelberg, Germany), plated on 0.5% gelatine-coated tissue culture dishes in Medium MV2 with C-39225 supplement mix (ECGMv2, Promo Cell). Cells were used between passages two and six. Endothelial cells from young adults were used for Figure 1, Figure 2 and Figure 3. Because of restricted availability, cells from juvenile donors were also used for the data in Figure 4, Figure 5, Figure 6, Figure 7 and Figure 8. The gene expression analysis by cDNA microarray was made with the endothelial cells from young adults and the analysis by RNA sequencing was performed with the cells from juvenile donors. No major differences were observed between cells from young and adult donors. HDMECs were transfected with a pool or single siRNAs against ZONAB (5′-AGACGUGGCUACUAUGGAA-3′ and 5′-CAACGUCAGAAAUGGAUAU-3′) or nontargeting siRNAs (5′-UGGUUUACAUGUCGACUAA and 5′-UGGUUUACAUGUUGUGUGA-3′). All siRNAs were obtained from Sigma-Aldrich (St. Louis, MO, USA) and were synthesised with dTdT 3′-overhangs. Depending on the experimental condition, HDMECs were plated on different types of dishes (e.g., 30,000 cells on 10 mm cover glasses in 48-well dishes and 8-well slides for electrical impedance measurements, cover glass coated with 0.8 mg/mL Growth factor-reduced Matrigel, Corning 354230; or 6-well dishes, 200,000 cells per well). The following day, transfection of siRNAs was performed using Lipofectamine RNAmax according to the manufacturer’s instructions (Thermo Fisher Scientific UK, Loughborough, UK). The medium was replaced 24 h later, and the cells were collected after 2–3 days of additional culture for further analysis. An ECIS impedance analyser Z-Theta (Applied Biophysics, Inc., Troy, NY, USA) was used to measure transendothelial electrical resistance. Measurements were performed for one hour (values were stable after about 5–10 min) at 500 Hz for transendothelial electrical resistance measurements and 64,000 Hz for capacitance measurements to control for electrode coverage.

### 2.2. Immunofluorescence and Protein Analysis

Cells were fixed either with methanol at −20 °C for 10 min or with 3% PFA for 20 min at room temperature. PFA-fixed cells were permeabilised with 0.3% Triton X-100 in PBS containing 0.3% BSA for 5 min. The cells were incubated overnight at 4 °C with primary antibodies. All primary antibodies used are listed in Table S10. The samples were then washed three times with PBS before incubating with fluorescent secondary antibodies (Table S10). For nucleus visualisation, Hoechst 33258 (Sigma-Aldrich) was used. Cells on coverslips were mounted with Prolong Gold mounting medium (Thermo Fisher Scientific, Waltham, MA, USA) and stored at 4 °C. Images were taken with a fluorescent microscope (Zeiss Axio Skop MOT2 microscope, Zeiss, Oberkochen, Germany) with a 40×/NA1.2 or 60×/NA1.4 oil immersion objectives using Simple PCI software (Version 3.1, Hamamatsu Photonics, Hamamatsu, Japan) or an inverted Nikon microscope (Eclipse Ti-E, Nikon, Tokyo, Japan) using a 60×/1.4 NA oil immersion objective lens, a CoolSNAP HQ2 camera (Photometrics, Tucson, AZ, USA), and Nikon software (NIS Elements, Version 5.22). Adobe Photoshop or Fuji/ImageJ (Version 2.16.0/1.54i, Build 26d66057dd) was used to adjust contrast. For immunoblotting of whole-cell lysates, the cells were washed twice with PBS, lysed in SDS-PAGE sample buffer, and denatured at 70 °C for 10 min [11]. The samples were then homogenised with a 1 mL syringe and a 25 g and 34 g needle before running on SDS-PAGE gels and transferring to PVDF or nitrocellulose membranes. The membranes were washed and blocked with 5% defatted milk powder dissolved in PBS containing 0.1% Tween-20. The used primary and secondary antibodies are detailed in Table S10.

### 2.3. Matrigel Plug Angiogenesis Assay

The Matrigel plug assay was performed and analysed as described previously (Birdsey et al., 2008 [20]). In brief, C57BL/6 mice were injected with a mix of Matrigel (BD), heparin, and, depending on the condition, FGF, and 2 μM siRNAs near the abdominal midline. Plugs were harvested after 7 days postmortem, fixed in 4% paraformaldehyde in PBS for 2 h at room temperature, transferred to 70% ethanol, embedded in paraffin, and processed for haematoxylin and eosin staining. The same siRNAs were used as for human cells (the corresponding sequences between human and mouse are 100% identical). For quantification, vessels contained in the Matrigel plug were identified by the presence of nucleated cells surrounding a lumen containing red blood cells. Vessels were counted in four fields of view using a 20× objective lens. Experiments were performed according to the Animals (Scientific Procedures) Act of 1986. 

### 2.4. Capillary-like Formation on Matrigel

Ice-cold growth factor-reduced Matrigel (BD Biosciences, Franklin Lakes, NJ, USA; 8–10 mg/mL, 50 μL/well) was added to flat-bottom 96-well plates and allowed to solidify for 1 h at 37 °C. HDMECs (15,000 cells/well) that had been transfected with siRNAs 48 h before were seeded in duplicates into the coated wells. The plates were then incubated at 37 °C in ECGMv2. Pictures were taken at different times, and capillaries were fixed in phosphate-buffered formalin at the end of the experiment. Branching points were quantified in four different fields and two different experiments per condition. Pictures were taken at different times using a 5×/0.12 NA objective lens on an inverted microscope (DMIRB; Leica, Wetzlar, Germany). 

### 2.5. Bromodeoxyuridine Incorporation Assay

HDMECs were cultured on glass coverslips and transfected with siRNAs. The ells were starved for 2 days to synchronise proliferation by using medium with one-tenth of the supplement. Complete ECGMv2 medium containing Bromodeoxyuridine (BrdU, 10 µM) was then added, and the cells were incubated for 4 or 8 h. The cells were then fixed in 100% methanol for 7 min at −20 °C, washed twice with PBS, and incubated with 1.5 M HCL for 30 min. After washing 4 times with PBS and blocking with 1% BSA and 0.1% NaN_3_ in PBS overnight, the cells were incubated with mouse antibodies specific for BrdU and DNase1 for 1 h. After washing three times with 1% BSA and 0.1% NaN_3_ in PBS, the cells were incubated with fluorescent secondary antibody and Hoechst dye for one hour. The samples were then washed and mounted onto glass slides with ProLong^®^ Gold Antifade Reagent (Thermo Fisher Scientific UK, Loughborough, UK). BrdU-positive cells were counted and quantified as a percentage of all cells per field of view.

### 2.6. Cell Trace CSFE

The day after RNA interference, the supplement of the medium was reduced to one-tenth, and, 24 h later, Cell Trace-DMSO stock was added at a final concentration of 1 μM for 20 min. Cells were then washed with complete ECGMv2 medium. Control cells for the baseline were collected immediately, and samples for measuring the effect on proliferation were collected 48 h later. Cells were collected in Trypsin EDTA, spun down at 1500 rpm for 5 min, and fixed in 3% PFA for 15 min. CSFE levels were measured by flow cytometry using a BD LSRFortressa^TM^ machine (BD Biosciences, Franklin Lakes, NJ, USA).

### 2.7. Cell Number 

To assess cell number, DNA content was measured. In brief, cells were plated in triplicate into 96-well plates at equal cell densities, and RNA inference was performed as described above. The medium was removed after three days, and samples were frozen at −80 °C. DNA contents were quantified using a CyQUANT Cell Proliferation Assay kit (Thermo Fisher Scientific) according to the manufacturer’s instructions.

### 2.8. Propidium Iodide Assay

Cell cycle analysis was performed by flow cytometry using propidium iodide. Cells were collected 72 h after transfection using trypsin for 10 min. They were then centrifuged and rinsed twice in PBS before fixation with cold 70% ethanol for 30 min on ice. Following fixation, the cells were centrifuged and rinsed twice with PBS. The PBS was removed after the final rinse, and 50 μL of RNase A solution in PBS (Roche 10109142001, Basel, Switzerland, 100 µg/mL, DNAse-free) was added to the pellet, followed by 400 µL of propidium iodide solution in PBS (Sigma P4170, St. Louis, MO, USA, 50 µg/mL). After a 5-min incubation at room temperature, the propidium iodide staining was measured using a BD LSRFortessa^TM^ X-20 Cell Analyser with BD FACS Diva software (Version 9.0; BD Biosciences). Data analysis was performed using Flowing Software (Version 2.5.1, Perttu Terho, Turku Centre for Biotechnology, Turku, Finland). At least 10,000 events (single cells positive for PI staining) were analysed per sample.

### 2.9. Quantitative Polymerase Chain Reaction (qPCR)

On ice, cDNA (50 ng/µL) was placed in quadruplicates into a MicroAmp Optical 96-well Reaction Plate (Thermo Fisher Scientific) with PowerUp SYBR Green Master Mix (Thermo Fisher Scientific) and 5 µM each of the two primers of interest (detailed in Table S11). A reaction volume of 10 µL was used. Fluorescence was measured using a Quant Studio 6 Flex Real-Time PCR System (Thermo Fisher Scientific). Relative mRNA levels were determined by standardising target primer fluorescence signals with the corresponding endogenous control fluorescence signals. Values were then normalised with the respective control values (Quant Studio 6 software was used). 

### 2.10. Senescence-Associated β-Galactosidase Assay

A quantity of 30,000 cells per well were plated in 48-well plates coated with 0.8 mL/mL Matrigel GFR. The following day, siRNA transfections were performed. After 3 days, the cells were washed in PBS, fixed in 3% paraformaldehyde (5 min, room temperature), washed again in PBS, and incubated at 37 °C overnight (without CO_2_) with senescence-associated β-Galactosidase staining solution (0.1% 5-bromo-4-chloro-3-indolyl b-D-galactosidase (X-Gal), 5 mM potassium ferrocyanide, 5 mM potassium ferricyanide, 150 mM NaCl, 2 mM MgCl_2_ in 40 mM citric acid/sodium phosphate, pH 6.0). For positive (lysosomal β-gal) and negative (bacterial β-gal) controls, the solution was brought to pH 4.0 or pH 7.5, respectively. After colour development, the cells were washed twice with PBS and once with methanol before examination with a brightfield Leica DM IL microscope (Leica, Wetzlar, Germany) fitted with a 20× objective. At least three images were captured at different locations for each coverslip, and senescence was determined by the percentage of SA-β-gal-positive cells per image.

### 2.11. Caspase and Necrosis Assays

The activities of caspases 3 and 7 were determined using the Apo-One^®^ Homogeneous Caspase 3/7 assay kit (Promega, Madison, WI, USA) according to the manufacturer’s instructions. The fluorescence of the samples was measured at 490 nm excitation and 530 nm emission with a FLUOstar OPTIMA plate reader (BMG LABTECH Inc., Durham, NC, USA). Necrosis was assessed by measuring lactate dehydrogenase release into the medium using the CytoTox-ONETM Homogeneous Membrane Integrity Assay (Promega, Madison, WI, USA), according to the manufacturer’s recommendations. The fluorescence of the samples was measured at 560 nm excitation and 590 nm emission with the FLUOstar OPTIMA plate reader.

### 2.12. ROS Assays

Total ROS was measured using 2′,7′-Dichlorodihydrofluorescein diacetate (DCFHDA; Merck Life Science UK Ltd., Dorset, UK) [21]. Cells, cultured and transfected with siRNAs in 96-well plates, were incubated with 10 µM DCFHDA in serum-free live cell imaging solution (Thermo Fisher Scientific) for 1 h. The medium was then removed and replaced with 50 µL of fresh live cell imaging solution. Fluorescence was then read with a plate reader at 485 nm excitation and 520 nm emission. The cells were then imaged with a Leica 4× lens for cell number measurements. Fluorescence measurements were then divided by cell numbers. To measure mitochondrial ROS, two sets of cells were plated in a 96-well plate in quadruplicate samples for each condition. The cells were then transfected with control or ZONAB siRNAs. After 3 days, one set of samples received MitoTracker Orange CM-H_2_TMRos (Thermo Fisher Scientific), which becomes fluorescent when oxidised. The second set of samples received MitoTracker Orange CMTMRos (Thermo Fisher Scientific). Both MitoTrackers were added at a concentration of 100 nM and were incubated for 10 min. Cells were washed in medium and then left for 10 min in fresh medium. Cells were washed again in medium to remove unincorporated MitoTracker reagents before fixation with 3% PFA in PBS. Fluorescence was then measured using a FLUOstar OPTIMA plate reader and BMG LABTECH software, version 3.1 (excitation 544 nm and emission 590 nm).

### 2.13. Seahorse Assays

Cells were seeded into a Seahorse XFe96/XF Pro Cell Culture Microplate (Agilent, Santa Clara, CA, USA) at a density of 0.7 × 10^4^ cells/well in 50 µL of ECGMv2 medium. The following day, the cells were transfected with siRNAs for 24 h followed by incubating in the fresh culture medium for 48–72 h. One hour before the Seahorse assay, the culture medium was replaced with Seahorse XF Assay Medium, composed of Seahorse XF DMEM Medium (pH 7.4, Agilent, Santa Clara, CA, USA) supplemented with 10 mM glucose, 1 mM pyruvate, 2 mM L-glutamine, and 1% C-39225 supplement (Agilent, Santa Clara, CA, USA). All experiments measuring energy metabolism were run with a Seahorse XF Pro Analyser using the software to run and analyse the experiments provided by the manufacturer (Agilent, Santa Clara, CA, USA). The mitochondrial stress test was run according to the manufacturer’s instructions using reagents purchased from Merck Life Science UK. Assay results were normalised by quantifying cell numbers using the CyQUANT assay. Briefly, cells in the Seahorse Cell Culture Microplates were frozen overnight at −80 °C. The CyQUANT assay was performed using the CyQUANT^TM^ Cell Proliferation Assay Kit (Invitrogen, Carlsbad, CA, USA) the next day, following the manufacturer’s guidelines. Fluorescence was measured at an excitation wavelength of 480 nm and an emission wavelength of 520 nm. The CyQUANT data were uploaded into the Seahorse Wave software (https://seahorseanalytics.agilent.com/; before 20 December 2025) for normalisation of the Seahorse experiments. 

### 2.14. Expression Analysis by Affymetrix cDNA Gene Expression Microarray and RNA Sequencing

Cells plated in 6-well dishes were transfected with either control or ZONAB siRNAs. After 4 days, total RNA was isolated using the RNeasy Mini Kit ((Qiagen, Venlo, The Netherlands). The RNA samples were then analysed by UCL Genomics using either an Affymetrix cDNA gene expression microarray or by RNA sequencing. Labelled gene chips were scanned, using a confocal argon ion laser (Agilent Technologies), and the data were analysed using Gene Spring 7.2 software (Agilent). RNA sequencing was also performed by UCL Genomics. Briefly, libraries were generated using a Watchmaker RNA sequencing kit with mRNA enrichment and unique dual indexing. Sequencing was performed with an Illumina NextSeq 2000 system (Illumina, Inc., San Diego, CA, USA). The data was analysed using the Galaxy Europe site (https://usegalaxy.eu/) for the differential expression analysis (with MultiQC, RNA STAR, htseq-count and DESeq2) using the Ensembl GRCh37 (hg19) annotation file and Galaxy ProteoRE (https://proteore.org/) for the functional and pathway analysis [22,23,24,25,26,27,28,29].

### 2.15. DNA Methylation Arrays

Cells plated in 6-well dishes were transfected with siRNAs the following day. After 4 days, genomic DNA from cells was extracted using a QIA amp DNA Mini and Blood Mini kit (Qiagen) according to manufacturer instructions. To identify methylated or unmethylated cytosines, DNA was treated with sodium bisulfite using the EZ DNA methylation kit (Zymo Research, Irvine, CA, USA) according to kit instructions. An Infinium^®^ Methylation EPIC Bead Chip (Illumina, Inc., San Diego, CA, USA) was used for the analysis at UCL Genomics Facility, using 45 µL of each sample containing 500 ng of DNA according to the kit instructions. Analysis was completed either with Illumina Genome Studio software (version 2.0.4) or with the R programme Bioconductor package, the Chip Analysis Methylation Pipeline (Champ). Using this package’s samples.idat, the data was loaded, normalised, and analysed for singular value decomposition (SVD), and statistically significant methylated regions (DMRs) were found as described (Morris et al., 2014 [30]). 

### 2.16. Image Analysis and Statistics

All image analysis and quantification of immunoblots were performed with either Image J/Fiji or Adobe Photoshop. Quantitative data are shown as datapoints and/or means +/− one standard deviation. The *n* number is provided in the respective figure legends and refers to mice in the in vivo studies in Figure 1 or independent biological repeat experiments in the cell-based assays, except as otherwise indicated in the figure legends. Statistical analysis was performed with JMP Pro (Versions 16, 17 and 18) or GraphPad Prism (Versions 9.5.0 and 10.6.0). The statistical significance of data pairs was assessed with two-sided *t*-tests and, in experiments requiring multiple comparisons, Tukey–Kramer HSD tests following an ANOVA test. Microarray and RNA sequencing data were analysed for differential expression of each gene using a Wald test (indicated as *p*-value in the Supplementary Tables). The *p*-values were then adjusted using the Benjamini–Hochberg method for multiple testing with an expected false discovery rate of *p* < 0.05 (indicated as *p* (Corr) or *p*-value adjusted for multiple testing, respectively, in the Supplementary Tables). Differentially expressed genes were filtered by the corrected/adjusted *p*-values, retaining only genes with values *p*_corr/adj_ < 0.05 for further analysis.

## 3. Results

### 3.1. Depletion of ZONAB Inhibits Endothelial Cell Migration and Angiogenesis

We first asked whether ZONAB is functionally relevant for endothelial angiogenesis. We established a loss-of-function model for ZONAB using human dermal microvascular endothelial cells (HDMECs). HDMECs form well-assembled and functional tight and adherens junctions [31]. We transfected the cells with control non-targeting or two ZONAB-specific siRNAs (termed 3 and 4). Immunoblotting confirmed efficient depletion of both ZONAB isoforms (ZONAB-A and ZONAB-B) by individual siRNAs, as well as a pool of both ZONAB siRNAs (Figure 1A). Transfection of either one of the two siRNAs did not affect transendothelial resistance (Figure 1B), indicating that the cells were still able to form functional barriers.

We next tested whether ZONAB depletion impacts the endothelial angiogenic potential in vitro by performing Matrigel tubulogenesis assays. ZONAB depletion reduced network formation and led to a ~50% decrease in node formation (Figure 1C). ZONAB is thus functionally important for endothelial tube formation on Matrigel in vitro.

We next asked whether ZONAB also regulates the endothelial angiogenic potential in vivo. We performed Matrigel plug assays in mice combined with siRNAs as we did previously for ZO-1 [28]. ZONAB siRNAs led to a more than 50% reduction in vessel formation when ZONAB siRNAs were co-injected with FGF and Matrigel, indicating that vessels failed to invade the FGF-rich matrix in vivo (Figure 1D). ZONAB is thus essential for angiogenesis in vitro and in vivo.

### 3.2. ZONAB Regulates the Actin Cytoskeleton

The actomyosin cytoskeleton is crucial for cell migration and angiogenesis. Hence, we tested whether ZONAB depletion affects the actomyosin cytoskeleton and junction formation. ZONAB-depleted cells formed striking thick F-actin bundles along the cell periphery instead of the normal junctional actin pattern, independent of whether the two siRNAs were transfected together or individually (Figure 2A and Figure S1A). These peripheral F-actin bundles also contained phosphorylated MLC (ppMLC). Myosin IIA was also enhanced along those F-actin fibres and at tricellular corners, whereas AKAP-12 still outlined the lateral membrane (Figure 2A). Thus, ZONAB depletion induced actomyosin reorganisation. The cells generally appeared more spread, reflecting the increased basal actomyosin activation, a process generally associated with cell spreading on rigid substrates. ZONAB depletion did not affect ZO-1 localisation but led to reduced junctional staining of other proteins that are at least partially associated with tight junctions. Staining for the scaffolding protein JACOP/paracingulin/CGNL1, the tight junction transmembrane protein claudin-5, and the regulator of tight junction assembly IQGAP1 were strongly reduced (Figure 2B and Figure S1A). In contrast, core adherens junction proteins such as VE-cadherin, β-catenin, and p120 catenin appeared normally localised (Figure 2C). The exception was vinculin, which moved to focal adhesions along the cell periphery, reflecting the increase in myosin activation along the induced peripheral F-actin bundles and suggesting increased tensile forces acting on focal adhesions (Figure 2C and Figure S1A). 

Protein expression of JACOP, claudin-5, IQGAP1, and GEF-H1, a Rho guanine nucleotide exchange factor known to interact with JACOP [31], was reduced, as determined by immunoblotting, in agreement with the immunofluorescent data, and was also reduced if either one of the two ZONAB siRNAs was transfected individually (Figure 2D,E and Figure S1B). JACOP was already reduced at the mRNA level, but claudin-5 mRNA expression was not significantly affected (Figure 2F). Overall, these data indicate that ZONAB depletion led not only to a reorganisation of the actomyosin cytoskeleton but also to disruption of junctional proteins known to interact with and/or regulate actin dynamics. Given the increase in peripheral actomyosin fibres and the vinculin redistribution to focal adhesions, it is likely that cells adhere more tightly to the substrate, inducing the observed increase in cell spreading and reduction in angiogenesis. 

To assess the stability of the ZONAB-induced F-actin reorganisation, we treated cells with stimuli known to promote F-actin remodelling. Figure 2G shows that high glucose (25 mM, 24 h) or TNFα (5 ng/mL for 5 h) led to induction of differently arranged stress fibres in endothelial cells compared to controls cells (see Figure 2A); however, the high glucose treatment of control cells was already similar to ZONAB-depleted cells in normal medium and did not further change in high glucose medium. The TNFα-induced striking F-actin reorganisation in control cells did not occur in ZONAB-depleted cells, but TNFα appeared to reduce some of the peripheral ZONAB depletion-induced F-actin bundles, indicating that TNFα-induced actin remodelling requires ZONAB expression. 

### 3.3. ZONAB Depletion Reduces Endothelial Cell Proliferation 

The microscopy data in Figure 2 and Figure 3 suggested that ZONAB-depleted cells had lower cell densities at the end of experiments (Figure 2A–C and Figure 3A). Quantification of cell numbers after 3 days of ZONAB depletion, when cells had reached confluence, by CyQUANT assay revealed that cell numbers were indeed reduced by about 25% (Figure 3B). No increases in apoptosis (Apo-ONE) or necrosis (Cyto-TOX) were detected, indicating that the cell number changes were not due to increased cell death (Figure 3C). 

Next, we measured proliferation by adding bromodeoxyuridine (BrdU) for 4 h during the last day of the experiment to label cells in S phase. ZONAB-depleted cells exhibited a strong decrease in the number of cells that incorporated BrdU (Figure 3D,E), indicating that ZONAB-depleted cells no longer entered S phase on the last day of incubation. We next measured cell division using a carboxyfluorescein succinimidyl ester (CFSE)-based cell-tracking dye that is incorporated into DNA and split equally between the two daughter cells upon mitosis, resulting in weaker signals as the cells divide. Control cells had a significantly weaker CFSE fluorescence than ZONAB-depleted cells, indicating that ZONAB-depleted cells had replicated less often than control cells (Figure 3F,G). Thus, ZONAB depletion reduced endothelial cell numbers and proliferation. Propidium iodide (PI) staining combined with flow cytometry indicated that ZONAB-depleted cells were largely arrested in the G0/G1 phase of the cell cycle (Figure 3H,I). Thus, ZONAB depletion arrested endothelial cell proliferation by halting the cell cycle at G0/G1 phase.

### 3.4. ZONAB Regulates the Expression of Central Cell Cycle Regulators 

We next employed an Affymetrix cDNA gene expression microarray and RNA sequencing to determine the effect of ZONAB depletion on gene expression in endothelial cells (Tables S1 and S2). The two independent approaches largely confirmed each other. ZONAB depletion resulted in the deregulation of genes encoding cell cycle regulators, such as cyclins and cell cycle kinases (CDKs), CDK inhibitors, cell cycle-regulating transcription factors, and genes required for DNA packing, centromere assembly, and chromosome separation (Figure 4A, Tables S3–S6; KEGG cell cycle pathway coverage of 41% with a log_2_ cutoff of |0.5|). Immunoblotting and/or RT-qPCR confirmed downregulation of multiple cyclins (Figure 4B,C). Similarly, PLK-1 was downregulated (Figure 4C–E). Immunoblotting further revealed downregulation of CDKs and upregulation of cell cycle inhibitors (Figure 4C,D). Hence, ZONAB depletion led to genome-wide changes in the expression of genes that regulate cell cycle progression. 

Given the inhibition of cell cycle progression observed upon ZONAB depletion (Figure 3I), we next tested expression and phosphorylation of the tumour suppressor retinoblastoma protein (Rb), which regulates G1/S phase transition. Rb protein expression was increased after ZONAB deletion, but phosphorylated Rb was decreased (Figure 4D,F). Thus, ZONAB is required for efficient Rb phosphorylation and, thereby, inactivation. Similarly, expression of the tumour suppressor p53 was increased in ZONAB-depleted cells, as well as expression of the cell cycle inhibitor p21 (Figure 4D,E). Hence, the pattern of gene expression changes indicated an inhibition of G1/S phase transition by induction of inhibitory mechanisms, which agreed with the cell cycle behaviour observed in Figure 3. 

Deregulation of cell cycle genes was not restricted to G1/S phase transition but also affected important regulators of G2/M phase transition, such as PLK-1 (Figure 4B–E). Expression of genes required for centromere formation, chromosome separation, and mitosis was generally reduced (Figure 4A). PLK-1 promotes CENP-A loading on centromeres [32]. ZONAB depletion not only reduced CENP-A expression but also promoted dispersal of the remaining centromere protein (Figure 4G–I).

ZONAB depletion thus deregulated the expression of genes guiding efficient progression at different steps of the cell cycle, indicating a general reduction in the proliferation potential of endothelial cells.

### 3.5. ZONAB Depletion Stimulates Cellular Senescence 

The RNA sequencing data indicated not only cell cycle arrest but also altered expression of markers associated with cellular senescence (Figure 5A; Table S7). In particular, the expression changes indicated induction of a SASP and inflammation. ZONAB depletion significantly upregulated mRNA of inflammatory genes, such as IL1A, ICAM1, SELE, and MMP10 (Figure 5A,B). Depletion of ZO-1 did not affect, or only modestly affected, those inflammatory genes (Figure 5B). Increased expression of ICAM-1 upon ZONAB depletion was confirmed by immunoblotting after transfection of pooled or individual ZONAB-targeting siRNAs (Figure 5C and Figure S1C). Hence, ZONAB-depleted endothelial cells display characteristics of a SASP, and are in an increased inflammatory state in addition to reduced proliferation. 

A key marker of senescent cells is senescence-associated β-galactosidase (SAβG) activity. We tested whether ZONAB-depleted cells exhibit increased expression of SAβG using a colourimetric staining. About 80% of ZONAB-depleted cells were positive for SAβG as opposed to less than 10% of control siRNA-transfected cells (Figure 5D,E). Hence, ZONAB-depleted cells entered cellular senescence.

Akt activation due to increased phosphatidyl inositol-3 kinase (PI3K) activity can induce cellular senescence due to p53 and p21 induction [33]. Both proteins were also induced by ZONAB depletion (Figure 4D,E). Akt-induced senescence is favoured by increased expression of NF1 (neurofibromin), which we also observed in ZONAB-depleted cells (Table S2) [34]. Therefore, we tested whether Akt was activated and whether inhibition of the pathway attenuated senescence. PI3K stimulates PDK-dependent phosphorylation of Akt at threonine 308 (T308) and can be inhibited with the small molecule inhibitor ZSTK474. In agreement, increased Akt phosphorylation at T308, and p53 and p21 protein expression, were reduced by PI3K inhibition with ZSTK474 in ZONAB-depleted cells (Figure 5F,G). PI3K can also be activated by phosphorylation of serine 473 (S473) by mTORCII and other kinases [35]; however, phosphorylation of S473 was not affected by ZONAB depletion (Figure S2). Thus, Akt activation upon ZONAB depletion and induction of p53 and p21 was PI3K-dependent. Strikingly, the PI3K inhibitor effectively reduced the percentage of senescent cells labelled by SAβG staining (Figure 5D,E). These data indicate that induction of cellular senescence in ZONAB-depleted endothelial cells is indeed driven by PI3K/Akt signalling.

### 3.6. ZONAB Depletion Stimulates Reactive Oxygen Species

Senescence can be induced by oxidative stress, which is associated with activation of the p53/p21 pathway by reactive oxygen species (ROS) [3,36,37]. Total cellular ROS content, as well as mitochondrial ROS levels, were indeed upregulated in ZONAB-depleted cells (Figure 6A,B). ROS and PI3K/Akt signalling are tightly linked and regulate each other [38]. Hence, we tested whether drugs that rescue senescence induced by H_2_O_2_, such as nicotinamide, a form of vitamin B3 that can also help mitigate senescence by influencing NAD^+^ levels, and N-acetylcysteine (NAC), an antioxidant that scavenges ROS, can rescue the induction of cellular senescence of ZONAB-depleted endothelial cells. Both nicotinamide and NAC strongly reduced the percentage of SAβG-positive ZONAB-depleted cells (Figure 5D,E). ZONAB depletion-induced senescence can thus be effectively rescued by drugs known to counteract ROS.

Our data indicate that the ZONAB depletion-induced increase in ROS is functionally relevant for cells to enter senescence. Increased ROS levels are expected to stimulate increased expression of the transcription factor Nrf2, which induces an anti-ROS response [39]. ZONAB depletion indeed stimulated increased Nrf2 expression and reduced levels of its inhibitor, Keap1 (Figure 6C–E). However, only one of its target genes tested, NQO1, was induced, whereas HO-1 (HMOX1) was repressed (Figure 6C–E). Thus, ZONAB depletion induced expression of Nrf2; however, induction of the key antioxidant gene HO-1 failed. 

HO-1 is required to prevent endothelial senescence, and overexpression alleviates endothelial senescence via a p53-dependent mechanism [40,41]. Hence, we further investigated possible mechanisms underlying HO-1 downregulation. Nrf2 cooperates with peroxisome proliferator–activated receptor gamma coactivator 1 alpha (PGC-1α) in the regulation of antioxidant genes [42]. RNA sequencing indeed indicated that ZONAB depletion led to decreased expression of PGC-1α (PPARGC1A) and key target genes in addition to HO-1, such as the mitochondrial transcription factor A (TFAM) (Tables S2 and S9). Immunoblotting confirmed the downregulation of PGC-1α (Figure 6C,D). Hence, the reduced expression of PGC-1α likely contributes to the failure of Nrf2 to induce HO-1.

To further understand the partial disruption of the Nrf2 pathway, we tested for SQSTM1/p62, which can activate Nrf2 and is itself a target gene of Nrf2 [43]. In agreement with the downregulation of Keap1, SQSTM1 phosphorylation increased upon ZONAB depletion, indicating activation (Figure 6C,D). SQSTM1 is an Nrf2 target gene and its mRNA expression was upregulated by RNA sequencing (Table S2); total protein remained unchanged, possibly reflecting increased autophagy and, hence, turnover of SQSTM1 (Figure 6C,D). 

Our data thus indicate that ZONAB depletion stimulates ROS and oxidative mechanisms; however, induction is incomplete, and Nrf2/PGC-1α signalling target genes crucial for the prevention of cellular senescence, such as HO-1, are repressed, as is PGC-1α itself. 

### 3.7. ZONAB Regulates Mitochondrial Fragmentation and Metabolic Activity

PGC-1α regulates the biosynthesis of mitochondria; hence, depletion of ZONAB may regulate the accumulation of mitochondria via PGC-1α repression [44]. Loss of the mitochondrial network and fragmentation can lead to mitochondrial dysfunction and senescence by excessive production of ROS [45,46]. In mammalian cells, mitochondrial mass and network formation is regulated by fusion and fission. Hence, we first asked if the mitochondrial network became fragmented upon ZONAB depletion. 

Dynamin-Related Protein 1 (DRP1) is key to the regulation of mitochondrial fission [47]. ZONAB depletion led to about twofold DRP1 upregulation (Figure 7A,B). ZONAB depletion altered the DRP1 distribution from a more filamentous to a more dispersed and punctate staining pattern, which displayed a reduction in branch length (Figure 7C,D and Figure S3A). Staining with a mitochondrial membrane potential-sensitive MitoTracker and anti-TOMM20 antibodies confirmed that mitochondria in ZONAB-depleted cells became more fragmented (Figure 7E–G and Figure S3B). TOMM20 protein expression was reduced, indicating less mitochondrial mass (Figure 7H,I). In addition to TFAM, several other mitochondrial genes and tricarboxylic acid cycle (TCA; Krebs cycle) components were downregulated upon ZONAB depletion, also indicating reduced mitochondrial biosynthesis (Table S8). The MitoTracker/TOMM20 ratio was increased, indicating that the remaining mitochondria were working at increased activity (Figure 7J). The reduced total mitochondrial capacity was confirmed by Seahorse mitochondrial stress test experiments that revealed that compensatory respiration (i.e., maximal activity) was almost halved by ZONAB depletion (Figure 7K). The basal activity was not affected by ZONAB depletion (Figure 7L). As mitochondrial mass was reduced by ZONAB depletion, the remaining mitochondria must thus work at a higher rate to achieve the same basal activity, which was supported by the increased MitoTracker/TOMM20 ratios (Figure 7J,L). Supporting mitochondrial function with nicotinamide, which attenuates senescence (Figure 5D,E), rescued much of the ZONAB depletion-induced drop in compensatory respiration, and nicotinamide-treated control and ZONAB-depleted cells had the same maximal mitochondrial activity (Figure 7K). These data support a model in which mitochondrial dysfunction in ZONAB-depleted endothelial cells drives induction of cellular senescence.

Increased DRP1 expression can lead to mitochondrial malfunction, increased ROS, and cellular senescence [48,49]. DRP1 can be inhibited by the small molecule inhibitor midivi-1 [50]. Hence, we tested whether DRP1 inhibition attenuated the entry into cell senescence of ZONAB-depleted cells. Figure 7M,N shows that midivi-1 strongly reduced the number of SAßG-positive cells. Thus, inhibition of DRP1 attenuates cellular senescence of ZONAB-depleted cells, further supporting the link between ZONAB depletion-induced DRP1 induction and cellular senescence.

### 3.8. ZONAB Depletion Induces DNA Methylation of Genes Involved in Cellular Senescence

Changes in DNA methylation have been shown not only to establish but to maintain and stabilise the cellular senescent phenotype [51,52]. DNA methylation is an epigenetic mechanism that contributes to silencing genes required for cell proliferation. To test if ZONAB depletion affects DNA methylation, we used bisulfite-converted genomic DNA from control and ZONAB-depleted endothelial cells to screen an Illumina genome methylation EPIC array. We detected hypermethylated cytosines in the PLK-1 gene promoter in ZONAB-depleted cells (Figure 8A), in agreement with the reduced expression described in Figure 4. Additionally, we identified several other differentially DNA-methylated regions in the promoters of genes required for proliferation, such as WRN Helicase Interacting Protein 1 (WRNIP1), methyltransferase-like protein 7A (METTL7A), and the cell cycle-regulating transcription factor forkhead box M1 (FOXM1) that were found to be downregulated in the gene expression analysis (Tables S2 and S9). Expression of genes such as FOXM1 is essential to prevent cellular senescence [53]. Hypermethylation of the FOXM1, WRNIP1, and METTL7A promoters correlated with decreased expression (Figure 8B–D). Thus, ZONAB depletion led to increased DNA methylation of genes whose repression leads to cell cycle arrest and cellular senescence.

## 4. Discussion

Our data demonstrate that ZONAB regulates endothelial cell proliferation and migration, and that its expression is required to prevent endothelial cells from entering cellular senescence. Hence, ZONAB depletion was found to disrupt the normal angiogenic behaviour in vitro and growth factor-induced angiogenesis in vivo. On a cellular level, we found that ZONAB regulates the expression of cell cycle regulators that are required for key steps along the cell cycle, and that ZONAB depletion led to the induction of epigenetic changes on promoters of genes involved in cell proliferation and senescence. In ZONAB’s absence, mitochondria became deregulated, and ROS was induced, but no complete oxidative stress response was induced. Entry into the senescent state induced by ZONAB downregulation could be prevented by drugs supporting mitochondrial function, inhibiting mitochondrial fragmentation and attenuating increased ROS levels or PI3K activation. Hence, mitochondrial deregulation and increased ROS production are linked to ZONAB depletion-induced cellular senescence of endothelial cells.

ZONAB-depleted endothelial cells have a reduced angiogenic potential in culture and in vivo. The in vitro assay is quick and is primarily affected by the effect of ZONAB depletion on the cytoskeleton; the in vivo assay spreads over days and is also affected by defects in proliferation. Senescent endothelial cells undergo a profound remodelling of the actin cytoskeleton, which includes actomyosin remodelling, redistribution of focal adhesions, and elevated traction forces, leading to less mobile cells that form more stable focal adhesions [54,55]. We observed similar cytoskeletal changes in ZONAB-depleted cells. Basal actomyosin activation, however, is unlikely to directly contribute to inhibition of proliferation, as such processes are generally rather associated with increased proliferation. As senescent cells also cease to proliferate, in vivo angiogenesis defects are likely to be direct consequences of ZONAB-depleted cells having entered cellular senescence.

The cytoskeletal remodelling of ZONAB-depleted cells also affected junctional proteins, most notably JACOP, an actomyosin-organising adaptor, and claudin-5, a major tight junction adhesion protein required for blood–brain barrier integrity [56]. Vinculin was removed from cell–cell junctions and redistributed to focal adhesions, which coincided with an analogous redistribution of active myosin-II and F-actin. Vinculin recruitment is a well-established indicator of increased tensile forces acting on focal adhesions and adherens junctions, respectively. Hence, the cytoskeletal phenotype indicates a reinforcement of forces acting on cell-substrate adhesions and a weakening of forces on adherens and tight junctions. Vascular cell senescence in vivo increases tight junction permeability and decreases blood–brain barrier function in aged mice [57]. We have not observed changes in transendothelial electrical resistance, and ZO-1 staining did not indicate tight junction disruption. Claudin-5 and, by RNA sequencing, claudin-12 were downregulated, whereas claudin-1 was induced (Table S2). Different pathological conditions have been reported to lead to claudin-1 induction in blood–brain barrier endothelia [58,59]; however, the functional consequences associated with claudin-1 expression are contradictory. It is also possible that the altered vinculin staining in senescent cells reflects cell junctions that are less stable; hence, the increased mechanical stress acting on endothelial cells in vivo may lead to a different permeability phenotype than in vitro.

Proliferation of ZONAB-depleted endothelial cells was strongly inhibited in agreement with the senescent phenotype. Cell cycle regulation was also observed in immortalised epithelial cell lines and tumour-derived cells from the kidney and gastrointestinal tract that did not become senescent; hence, ZONAB’s role in proliferation may lead to senescence but also regulates proliferation in non-senescent cells [9,10,11,15,18,58,60,61,62,63]. Similarly, in vivo data support a role of ZONAB in cell proliferation in different epithelial cell types [61,64,65]. A common function of ZONAB is thus the regulation of cell proliferation; however, its depletion in primary endothelial cells not only inhibits proliferation but to the induction of cellular senescence.

We had previously identified cyclin D1 and PCNA as ZONAB-regulated genes in epithelial cells, two genes that have been associated with ZONAB signalling in different epithelial cell types [10,11,62,65]. Cyclin D1 was also strongly downregulated in ZONAB-depleted endothelial cells, but the effect on PCNA was very modest. However, we have now identified a range of major cell cycle genes that were affected by ZONAB depletion in primary endothelial cells, including cyclins and kinases affecting different steps of the cell cycle; hence, ZONAB depletion has a broad effect on cell cycle regulation.

Some of the new ZONAB-regulated cell cycle genes identified here are involved in the regulation of endothelial proliferation, angiogenesis, and senescence. For example, cyclin A2 plays a crucial role in activating PLK-1, which is essential for cell proliferation [66]. Both cyclin A2 and PLK-1 are downregulated during cell senescence [67,68], and silencing PLK-1 in endothelial cells reduces tube-formation, implicating PLK-1 in the regulation of angiogenic behaviour [69]. Hence, both genes are ZONAB signalling targets important for angiogenesis. 

It is unlikely that all the genes deregulated upon ZONAB depletion are direct targets of ZONAB-regulated transcription. We discovered here that ZONAB signalling regulates the methylation of some of the genes that are downregulated or upregulated upon its depletion. For instance, the *PLK-1* promoter, which is hypermethylated in ZONAB-depleted cells, also becomes hypermethylated in response to ROS, leading to reduced *PLK-1* expression and cell senescence [70]. DNA methylation patterns in genes related to endothelial senescence are known to impact endothelial cell function and to contribute to vascular ageing and related diseases [71]. Senescence-associated DNA methylation often involves global hypomethylation (decreased methylation across the genome) and focal hypermethylation (increased methylation at specific gene locations) [72]. We identified several genes linked to cell proliferation and senescence that became hypermethylated in response to ZONAB depletion. These included genes associated with ageing, such as *WRNIP1*, *FOXM1*, and *METTL7A* [73,74,75]. *PARD3* induction was suggested to play a role as a biomarker for cell senescence [76]. Our results thus uncover a novel role of ZONAB in regulating DNA methylation and, thereby, the regulation of genes important for entry into cell senescence.

While downregulation of the cell cycle is a normal feature of cell senescence, cell cycle inhibition is a process that generally does not lead to entry into senescence. Hence, ZONAB depletion in endothelial cells must induce changes in addition to cell cycle inhibition. A common stimulus leading to cell senescence is increased ROS and oxidative stress. ZONAB depletion not only led to an increase in ROS levels but also to dysregulation of mitochondria, which is known to contribute to ROS induction. Our data further indicate that ZONAB regulates the expression of PGC-1α. PGC-1α regulates mitochondrial biosynthesis and, together with Nrf2, the expression of HO-1. Even in the presence of increased Nrf2 expression, ZONAB depletion led to PGC-1α and HO-1 repression and, therefore, failed ROS elimination, triggering cellular senescence. ROS stimulates senescence via a p53/p21-dependent mechanism [3,36,37]; ZONAB depletion indeed stimulated expression of both p53 and p21.

ROS induces PI3K/Akt [38]. We have observed a strong increase in phosphorylation of the PI3K substrate site of Akt, and inhibition of senescence in PI3K inhibitor-treated ZONAB-depleted endothelial cells. The most studied inducer of cell senescence is H_2_O_2_, which directly increases ROS and which can be rescued by nicotinamide or NAC [77,78]. With both drugs, we were also able to rescue ZONAB-depletion-induced senescence in endothelial cells, further supporting the conclusion that the increase in ROS was responsible for the induction of senescence of ZONAB-depleted endothelial cells.

In addition to the repression of PGC-1α and HO-1, we observed a reduction in mitochondrial mass and total respiratory capacity, which required remaining mitochondria to be more active. Mitochondrial dysregulation stimulates ROS production. ZONAB depletion led to increased expression of DRP-1 and, consequently, increased mitochondrial fragmentation. Entry into senescence was inhibited by a DRP-1 inhibitor or by stimulating mitochondrial function with nicotinamide. Hence, the regulation of mitochondria downstream of ZONAB signalling plays a functionally important role in endothelial cell homeostasis. 

## 5. Conclusions

ZONAB’s function in the regulation of endothelial cell homeostasis points to a possible role in vascular health. Endothelial cell senescence is thought to contribute to the development and/or progression of diseases such as atherosclerosis and microvascular conditions affecting the heart or brain, as well as neurodegenerative disorders such as Alzheimer’s disease [1,2,79]. Genome-wide genetic studies have shown that the ZONAB gene is associated with loci with single-nucleotide polymorphisms that increase the risk for developing atherosclerosis as well as high systolic blood pressure [4,5,6]. While it is currently unknown if and how those SNPs affect ZONAB function and/or expression, these observations, combined with our data, suggest that ZONAB malfunction may drive serious vascular diseases by inducing cellular senescence. Similarly, a recent study identified ZONAB/YBX3 as a transcription factor that interacts with enhancer sequence elements of alleles identified as risk loci for Alzheimer’s disease [80]. Hence, disruption of the here-identified role of ZONAB in regulating endothelial angiogenic behaviour and cellular senescence may promote common vascular and neurodegenerative diseases. Establishment of the necessary disease models will be required to test the pathophysiological importance of ZONAB in vivo or, rather, its downregulation in the development of such endothelial diseases, and to elucidate possible causal links between ZONAB deregulation and the development of vascular disorders.

## Data Availability

The RNA sequencing data have been submitted to the European Nucleotide Archive (accession number: PRJEB107969), and the microarray data to ArrayExpress (cDNA array: E-MTAB-16706; methylation array: E-MTAB-16704). All other data are available from Maria S. Balda (m.balda@ucl.ac.uk) or Karl Matter (k.matter@ucl.ac.uk).

## Supplementary Materials

The following supporting information can be downloaded at: https://www.mdpi.com/article/10.3390/cells15111015/s1, Figure S1: Deconvolution of ZONAB siRNAs. Figure S2: ZONAB depletion does not affect Akt phosphorylation at serine 473. Figure S3: ZONAB depletion induces increased mitochondrial fragmentation. Table S1: Affymetrix cDNA analysis—2-fold up- and downregulated genes by ZONAB depletion in HDMECs; Table S2: RNA sequencing expression analysis of significantly up- and downregulated genes by ZONAB depletion in HDMECs; Table S3: KEGG pathways relevant for endothelial homeostasis and angiogenesis, including lists of genes affected by ZONAB depletion (based on RNA sequencing data); Table S4: KEGG pathway enrichment analysis (based on RNA sequencing data); Table S5: GO cellular compartments significantly affected by ZONAB depletion (based on RNA sequencing data); Table S6: GO biological processes affected by ZONAB depletion (based on RNA sequencing data); Table S7: Cell cycle and senescence genes that are components of the respective KEGG pathways and that are deregulated by ZONAB depletion (based on RNA sequencing data); Table S8: Mitochondrial and TCA/Krebs cycle components deregulated by ZONAB depletion (based on RNA sequencing data); Table S9: Differentially methylated region (DMR) hits from Illumina EPIC array; Table S10: Source and dilution of primary and secondary antibodies used: Table S11: Sequence of primers for qPCR.
