# Supplementary material for: ZONAB Regulates DNA Methylation, Mitochondrial Function, and Entry into Cell Senescence of Endothelial Cells
Source: Cells. 2026 May 31;15(11):1015. doi: 10.3390/cells15111015 (PMC13256303; doi:10.3390/cells15111015)
Supplement: Supplementary file 1 [file cells-15-01015-s001.zip › cells-4213213-supplementary/Supplementary files/Supplementary Figures.pdf]

Supplementary Figures

ZONAB regulates DNA methylation, mitochondrial function and entry into cell senescence of endothelial cells

Wenyi Jiang, Eleanor Lynam, Juliette Delafosse, Graeme M. Birdsey, Anna M. Randi, Karl Matter, and Maria S. Balda

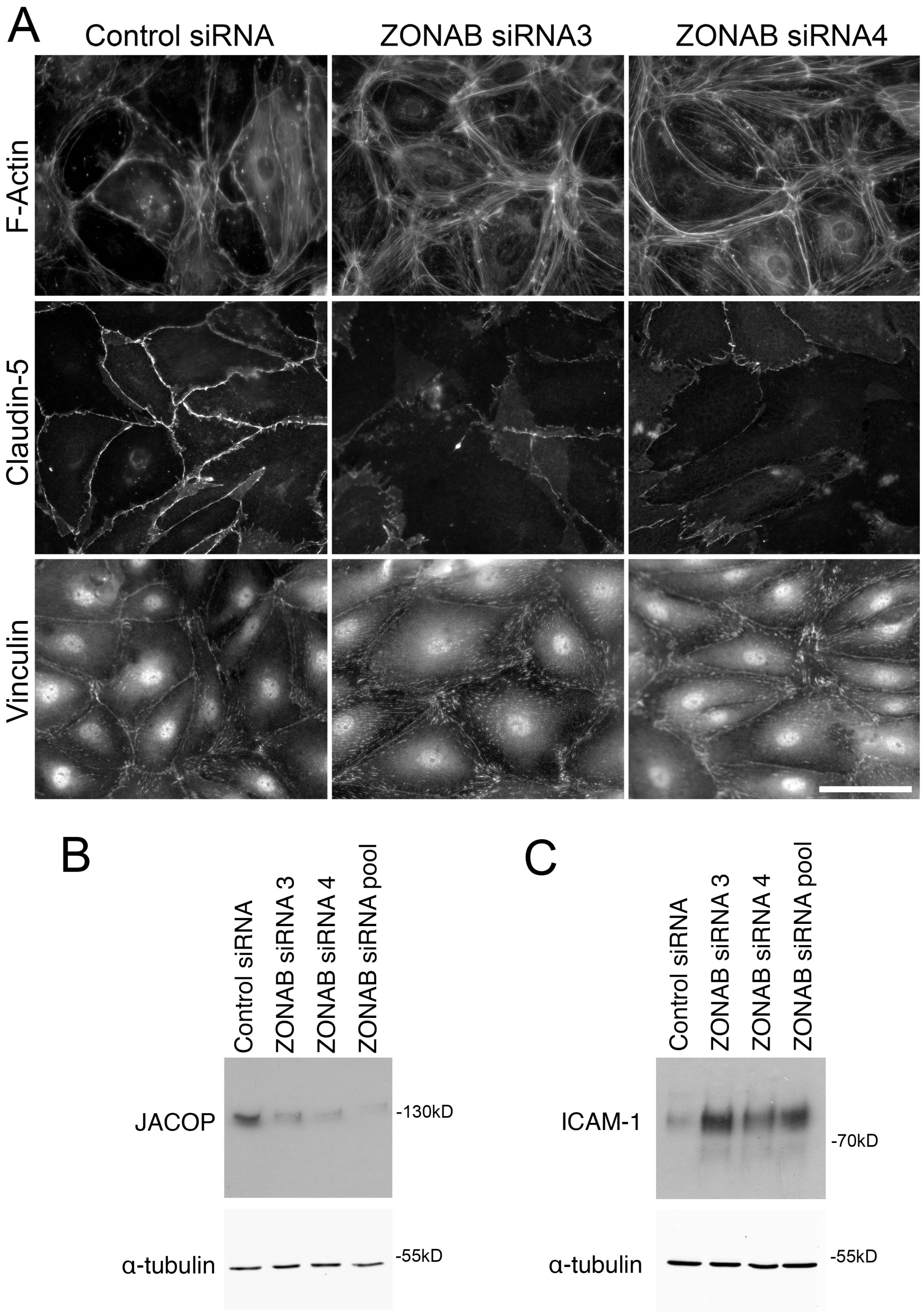

Figure S1. Deconvolution of ZONAB siRNAs (**A**) Endothelial cells on coverslips were transfected with control or individual ZONAB-targeting siRNAs. After 4 days, the cells were fixed and processed for fluorescent microscopy using either fluorescently labelled phalloidin to stain F-Actin, or antibodies against Claudin-5 or vinculin followed by fluorescent secondary antibodies. Scale bar, 50 μm. **(B, C)** Cell extracts generated from endothelial cells transfected with control or the indicated ZONAB-targeting siRNAs were analysed by immunoblotting with antibodies against JACOP, ICAM-1, or a α-tubulin.

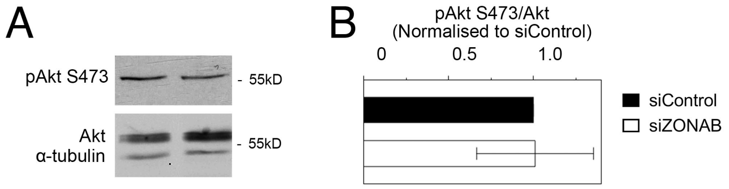

Figure S2. ZONAB depletion does not affect Akt phosphorylation at serine 473 (**A**) Endothelial cell extract derived from control or ZONAB siRNA-transfected cells was analysed by immunoblotting with antibodies specific for Akt phospho-serine 473, Akt, or α-tubulin. (**B**) Quantification of Akt S473 phosphorylation. Values were normalised to control siRNA-transfected cells (mean ± 1 SD, n=3).

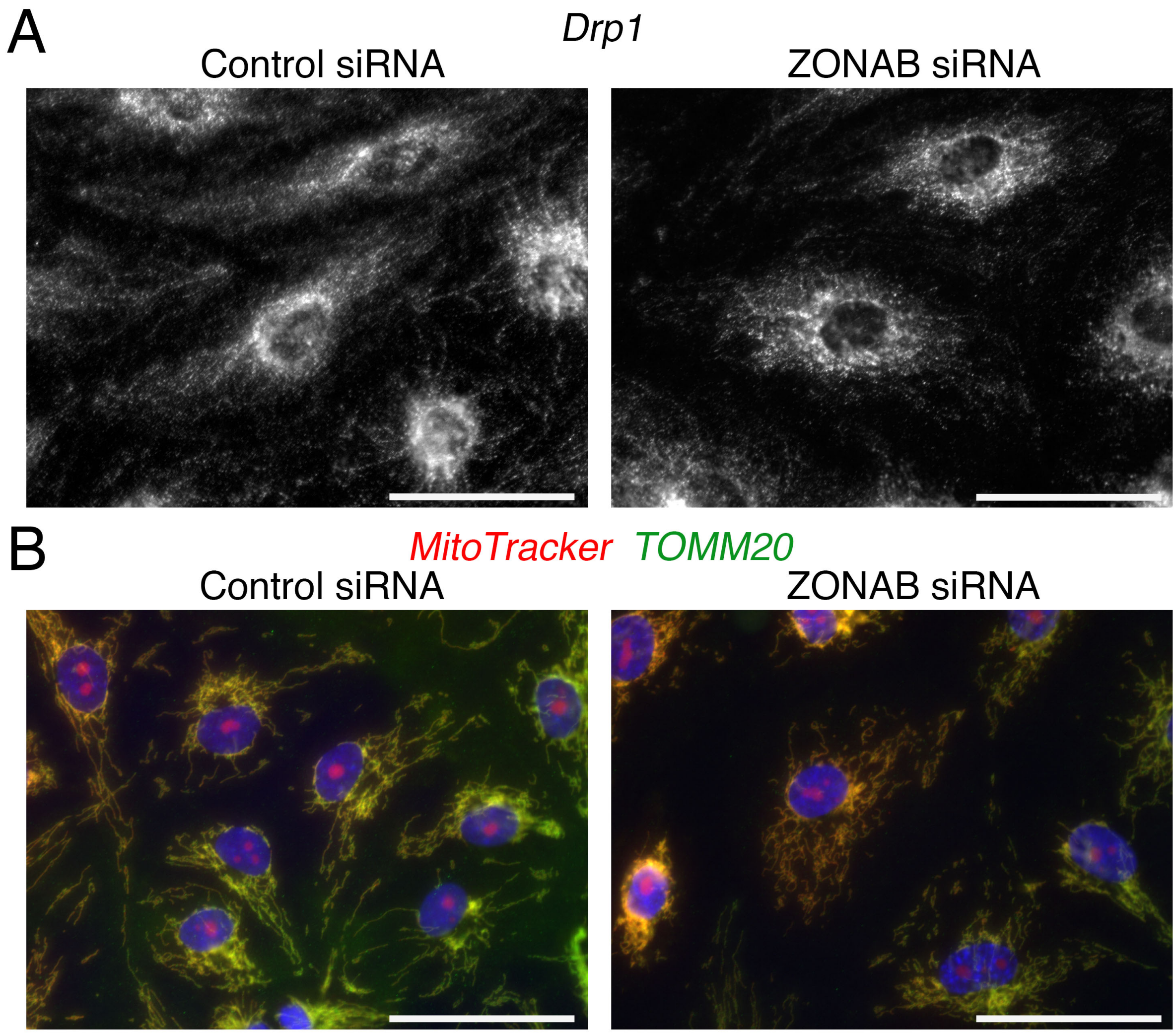

Figure S3. ZONAB depletion induces increased mitochondrial fragmentation. Shown are higher-resolution images of the panels shown in Figure 7. Panel A corresponds to Figure 7C, and Panel B to Figure 7E.
